# *Fusobacterium nucleatum* Bacteremia Presenting as Isolated Superior Mesenteric Vein Thrombophlebitis

**DOI:** 10.1155/2024/5349136

**Published:** 2024-06-14

**Authors:** Alaukika Agarwal, Ekrem Yetiskul, Ronak Patel, Faris Qaqish, Hamzah Qandil, Neville Mobarakai

**Affiliations:** ^1^Department of Internal Medicine, Staten Island University Hospital, 475 Seaview Avenue, Staten Island, NY 10305, USA; ^2^Department of Internal Medicine, SUNY Downstate Medical Center, 450 Clarkson Avenue, Brooklyn, NY 11203, USA

## Abstract

*Fusobacterium nucleatum* (*F. nucleatum*) is a commensal Gram-negative anaerobic bacterium that lives in the oral cavity and gastrointestinal tract of humans. While it is a regular resident of the human oral cavity, *F. nucleatum* has been implicated in various infections and inflammatory conditions. This case report highlights an unusual association between *F. nucleatum* and isolated superior mesenteric vein (SMV) thrombosis.

## 1. Introduction

Mesenteric vein thrombophlebitis can be triggered by hypercoagulable, malignant, or septic conditions. Although its occurrence due to septic conditions is rare, the associated mortality, particularly when diagnosed late, can exceed 30% [1]. *F. nucleatum*, a spindle-shaped rod with a Gram-negative classification, is frequently found in the oral flora. While traditionally not viewed as a pathogenic species within the oral cavity, it is increasingly recognized for its involvement in promoting inflammation, a well-established risk factor for thrombosis [2]. As a pathogen, *Fusobacterium* species typically causes oropharyngeal infections and can lead to septic thrombophlebitis of the internal jugular vein, known as Lemierre's syndrome [3]. Although rare, *Fusobacterium* has been documented as a complicating factor in intraabdominal infections, resulting in septic thrombophlebitis of the portal vein or one of its tributaries, known as pylephlebitis, with an incidence of 0.37–2.7 cases per 100,000 person-years [4]. In this case report, we present a case of septic SMV thrombophlebitis *with F. nucleatum* isolated on blood cultures in a 64-year-old male patient who presented to the hospital with nine days of intermittent periumbilical pain, fevers, chills, myalgia, and decreased appetite.

## 2. Case Presentation

We present the case of a 64-year-old male with a past medical history of hypertension, hyperthyroidism, gastroesophageal reflux disease, diverticulosis, prostate cancer status post radical prostatectomy eight years prior, and cholecystitis status post cholecystectomy one prior who presented to the emergency department with nine days of intermittent periumbilical abdominal pain. The pain was not associated with meals or bowel movements, and the patient denied any specific triggering event or consumption of new or unique foods. Accompanying symptoms included fevers, chills, myalgias, and poor appetite. He denied nausea, vomiting, diarrhea, constipation, shortness of breath, chest pain, dizziness, lightheadedness, melena, or hematochezia. A colonoscopy performed two years prior to presentation revealed multiple benign polyps.

Upon arrival in the emergency department, the patient's vital signs were recorded as follows: temperature of 98.5°F, heart rate ranging from 114 to 125 bpm, blood pressure of 111/75 mmHg, respiratory rate of 18 breaths per minute, and oxygen saturation of 99% on room air. Physical examination revealed a soft, nontender, and nondistended abdomen without guarding, organomegaly, and an absent Murphy's sign.

**Figure 1:**
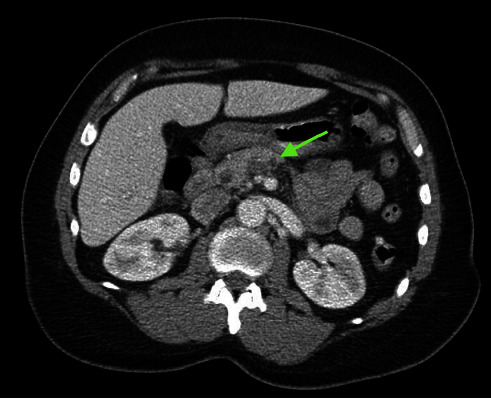
Axial section obtained from CT of the abdomen and pelvis showed findings consistent with superior mesenteric vein thrombophlebitis (green arrow).

**Figure 2:**
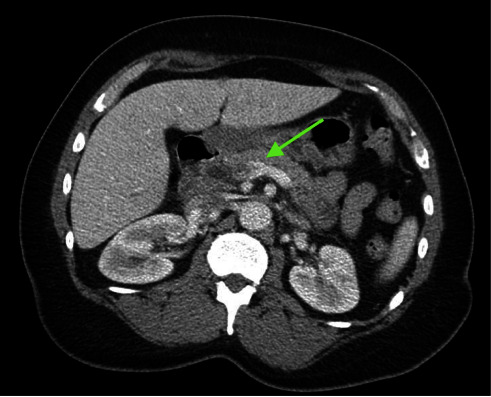
Axial section obtained from CT of the abdomen and pelvis with intravenous contrast demonstrates superior mesenteric vein thrombosis with surrounding inflammation consistent with thrombophlebitis (green arrow).

**Figure 3:**
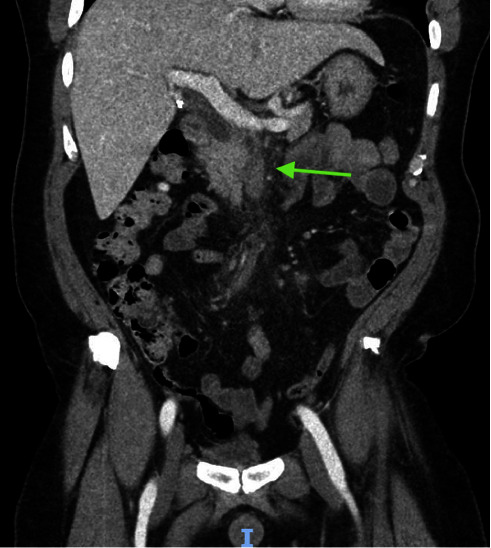
Coronal section obtained from CT of the abdomen and pelvis with intravenous contrast demonstrates superior mesenteric vein thrombosis with surrounding inflammation consistent with thrombophlebitis (green arrow).

Laboratory findings revealed a white blood cell count of 15.7 K/*μ*L (reference range: 4.8–10.8 K/*μ*L), total bilirubin of 2.1 mg/dL (reference range: 0.2–1.2 mg/dL), alkaline phosphatase of 160 U/L (reference range: 30–115 U/L), aspartate aminotransferase (AST) of 55 U/L (reference range: 0–41 U/L), and alanine aminotransferase (ALT) of 61 U/L (reference range: 0–41 U/L). Computed tomography (CT) of the abdomen and pelvis with intravenous contrast was performed to elucidate further the source of the patient's abdominal pain, which demonstrated postcholecystectomy changes with stable mild biliary ductal dilatation likely secondary to cholecystectomy status and superior mesenteric vein thrombosis with surrounding inflammation consistent with thrombophlebitis (Figures 1–3). Given the presence of mesenteric thrombophlebitis, therapeutic apixaban, empirical ceftriaxone, and metronidazole were started.

Further diagnostic evaluation during the patient's hospital course included hypercoagulable and malignancy screening. Thrombophilia evaluation demonstrated the absence of Antithrombin III deficiency, Protein C deficiency, Protein S deficiency, Prothrombin G20210A mutation, and Factor V Leiden. Antiphospholipid testing was also negative. The patient received his first dose of therapeutic apixaban after the hypercoagulable evaluation. Carcinoembryonic antigen, Ca 19-9, Ca-125, and alpha-fetoprotein were unremarkable. Blood cultures drawn on admission yielded growth of *Fusobacterium nucleatum*, and antibiotics were continued. Over the next six days, the patient's abdominal pain resolved, and the liver enzymes and total bilirubin levels improved. The patient was discharged on apixaban for a 6-month duration and amoxicillin-clavulanate to complete the course of antibiotics initiated in the hospital.

## 3. Discussion

The association between *Fusobacterium nucleatum* and SMV thrombophlebitis is a rare but clinically significant manifestation. The *Fusobacterium* genera are anaerobic residents of the human gut. Lemierre syndrome, characterized by infection of the oropharyngeal membranes and internal jugular vein thrombosis, is famously associated with *Fusobacterium necrophorum*, a sister species of *Fusobacterium nucleatum* [6]. While *F. necrophoru*m is the most common pathogen in Lemierre syndrome*, F. nucleatum* may also be involved [2, 6]. This species was discovered as a causative pathogen in a 19-year-old African-American male with thrombophlebitis of the external jugular vein [7]. However, gastrointestinal manifestation with portal vein thrombosis or SMV thrombosis remains a lesser-known association.

One case report describes a gastrointestinal variant of Lemierre syndrome, detailing *F. nucleatum* bacteremia-associated hepatic vein thrombosis in a 73-year-old male [8]. This pylephlebitis was likely associated with an intraabdominal infection. A second case report highlights the presentation of a 60-year-old Hispanic man with a 3-week history of fevers, drenching sweats, 15 lbs of unintentional weight loss, and intermittent epigastric abdominal pain. This patient was treated with piperacillin-tazobactam for *F. nucleatum* bacteremia associated with pylephlebitis and anticoagulation for six months [9]. The literature review also reveals a case of *Fusobacterium* bacteremia in a 59-year-old male who presented with back pain and was found to have acute inferior mesenteric vein thrombosis [10]. However, the authors describe *Fusobacterium* bacteremia without specifying a particular species. While these cases draw an intricate interplay between this species and portal vein thrombosis, no case reports to date narrate *F. nucleatum* bacteremia with isolated SMV thrombophlebitis.

The proinflammatory properties of *F. nucleatum* are yet to be established; however, proposed mechanisms suggest that these bacteria can directly activate coagulation pathways (factor XII), promote platelet aggregation, and induce vascular inflammation [11]. This pathway has not been explored in the literature and needs further study. It appears that while the oral and gastrointestinal areas are the primary point of access for these bacteria [11], it initiates entry and thrombosis at distant sites, implying that the underlying dissemination was likely systemic leading to an eventual localized thrombosis.

SMV thrombophlebitis carries an increased risk of morbidity and mortality and most often involves intraabdominal suppuration [12]. Our case exhibits the classical intermittent periumbilical pain associated with SMV thrombosis in a 64-year-old male. He also presented with fevers, myalgia, and decreased appetite, which encompasses a wide range of etiologies. CT imaging revealed superior mesenteric vein thrombosis with surrounding inflammation, which led to a diagnosis of SMV thrombophlebitis, which was further established with the help of clinical and laboratory findings.

The pathophysiological consideration remains the proinflammatory characteristic of *F. nucleatum* [13], a well-established risk factor for thrombosis not limited to the jugular vein as in Lemierre syndrome. The oral and gastrointestinal areas remain the primary access route for these bacteria, initiating entry and thrombosis at distant sites [14].

It is also important to consider the therapeutic approach involving such cases. There are no clear guidelines that expand upon the duration of anticoagulation for these patients. While prompt initiation of anticoagulation led to a favorable outcome for our patient, the preferred duration of anticoagulation remains unclear. In the absence of hypercoagulable and gastrointestinal malignancy markers, this patient was treated with anticoagulation for six months. The absence of these markers also reinforces the interplay of *F. nucleatum* with significant thrombotic events.

## 4. Conclusion

We highlight an unusual association between *F. nucleatum* and isolated SMV thrombophlebitis, which enhances the thrombotic nature of this species. Early identification and prompt therapy are crucial for favorable patient outcomes, but the guidelines surrounding the duration of anticoagulation remain unclear in the absence of hypercoagulable or malignant states.

## Data Availability

Data sharing is not applicable to this article as no new data were created or analyzed in this study.
